# Cardiac MRI in diagnosis, prognosis, and follow-up of hypertrophic cardiomyopathy in children: current perspectives

**DOI:** 10.1093/bjr/tqae033

**Published:** 2024-02-08

**Authors:** Tessa O M Spaapen, Anneloes E Bohte, Martijn G Slieker, Heynric B Grotenhuis

**Affiliations:** Department of Paediatric Cardiology, University Medical Centre Utrecht/Wilhelmina Children's Hospital, Heidelberglaan 100, 3584 CX, Utrecht, The Netherlands; Department of Radiology and Nuclear Medicine, University Medical Centre Utrecht/Wilhelmina Children's Hospital, Heidelberglaan 100, 3584 CX, Utrecht, The Netherlands; Department of Paediatric Cardiology, University Medical Centre Utrecht/Wilhelmina Children's Hospital, Heidelberglaan 100, 3584 CX, Utrecht, The Netherlands; Department of Paediatric Cardiology, University Medical Centre Utrecht/Wilhelmina Children's Hospital, Heidelberglaan 100, 3584 CX, Utrecht, The Netherlands

**Keywords:** Hypertrophic cardiomyopathy (HCM), Cardiac magnetic resonance imaging (CMR), echocardiography, clinical assessment, peadiatric, childhood-onset

## Abstract

Hypertrophic Cardiomyopathy (HCM) is an inherited myocardial disease characterised by left ventricular hypertrophy, which carries an increased risk of life-threatening arrhythmias and sudden cardiac death. The age of presentation and the underlying aetiology have a significant impact on the prognosis and quality of life of children with HCM, as childhood-onset HCM is associated with high mortality risk and poor long-term outcomes. Accurate cardiac assessment and identification of the HCM phenotype are therefore crucial to determine the diagnosis, prognostic stratification, and follow-up. Cardiac magnetic resonance (CMR) is a comprehensive evaluation tool capable of providing information on cardiac morphology and function, flow, perfusion, and tissue characterisation. CMR allows to detect subtle abnormalities in the myocardial composition and characterise the heterogeneous phenotypic expression of HCM. In particular, the detection of the degree and extent of myocardial fibrosis, using late-gadolinium enhanced sequences or parametric mapping, is unique for CMR and is of additional value in the clinical assessment and prognostic stratification of paediatric HCM patients. Additionally, childhood HCM can be progressive over time. The rate, timing, and degree of disease progression vary from one patient to the other, so close cardiac monitoring and serial follow-up throughout the life of the diagnosed patients is of paramount importance. In this review, an update of the use of CMR in childhood HCM is provided, focussing on its clinical role in diagnosis, prognosis, and serial follow-up.

## Introduction

Hypertrophic Cardiomyopathy (HCM) is a common inherited myocardial disease that can manifest at any age.^1^ HCM is characterised by hypertrophy of the left ventricular (LV) myocardium in the absence of abnormal loading conditions.^1–4^ The prevalence of HCM in children differs significantly from the adult population (1 in 500 individuals)^2^^,^^5^ and is estimated between 0.24 and 0.47 per 100 000 children. Despite its rare prevalence in childhood, HCM forms the second most common cardiomyopathy presenting in children as it accounts for about 42% of all paediatric cardiomyopathy cases.^3^^,^^6–8^ HCM is a clinically and genetically heterogeneous disorder, with a well-described phenotype ranging from asymptomatic to progressive heart failure or even sudden cardiac death (SCD).^2^^,^^5^^,^^9^^,^^10^ HCM carries an increased risk of life-long morbidity and mortality and is a leading cause of SCD, especially in children and young adults.^3^^,^^5^^,^^11^

Childhood HCM is characterised by three peaks of presentation; during infancy, childhood, and adolescence.^6^^,^^12^^,^^13^ Early-onset HCM manifests itself within the first year of life with often progressive myocardial hypertrophy and associated circulatory failure. Infants with HCM have the broadest spectrum of causes and the poorest prognosis compared to older children. Childhood-onset HCM is associated with a high risk for life-threatening ventricular arrhythmias combined with poor long-term outcomes.^6^^,^^12^^,^^14^ Manifestation of HCM during adolescence is similar to the adult type of HCM, with an increased risk for atrial fibrillation and heart failure, but a lower risk for SCD compared to younger patients.^6^^,^^15^^,^^16^ In contrast to adult HCM, the underlying aetiology of childhood HCM represents a more heterogeneous group of disorders, including mutations in cardiac sarcomeric genes, metabolism disorders, neuromuscular diseases, malformation syndromes, and mitochondrial disease.^4^^,^^8^^,^^11^^,^^17^ The age of presentation and the underlying aetiology therefore have a significant impact on the individual prognosis of children with HCM,^11^^,^^14^^,^^18^ as the highest risk of mortality is seen in infants.^8^^,^^10^ Accurate cardiac assessment is therefore crucial for the determination of diagnosis, prognosis and follow-up in especially the young HCM population.

Echocardiography is routinely used to perform cardiac evaluation and assess the degree of hypertrophy and cardiac function in HCM.^3^ Despite its non-invasiveness, low costs, and ready availability, echocardiography has several technical limitations including a reduced acoustic window, especially in the older child. Myocardial tissue characterisation can also not be performed, which may prohibit the distinction between HCM and, for example, an athlete’s heart.^3^^,^^4^^,^^19–22^ In contrast, cardiac magnetic resonance (CMR) provides significant advantages given its high spatial and temporal resolution, sharp contrast and three-dimensional (3D) tomographic images, allowing for a comprehensive cardiac evaluation of morphology, function, flow, and myocardial tissue characterisation.^8^^,^^19^^,^^23^^,^^24^ Nevertheless, the application of CMR is more time-consuming and more expensive compared to echocardiography, while motion artefacts may occur especially in young children as remaining still during scanning can be challenging.^3^^,^^23^ In addition, the specific risks of sedation or anaesthesia—typically required in children below the age of 10 years and with increased risk in more severe HCM—need to be weighed against the benefits of performing CMR as part of the diagnostic process.^25^ Each imaging modality therefore has its strengths and limitations. Overall, CMR may help with risk stratification for life-threatening ventricular arrhythmias and SCD, and as such, the appropriate use of CMR may have a beneficial impact on early diagnosis and improved survival.^4^^,^^5^ An integrated approach with a combination of both modalities would provide an optimal imaging strategy for children with HCM.

This review aims to provide an update on the use of CMR in childhood HCM, focussing on its role for diagnosis, prognosis, and follow-up.

## CMR imaging techniques

**Figure 1.**
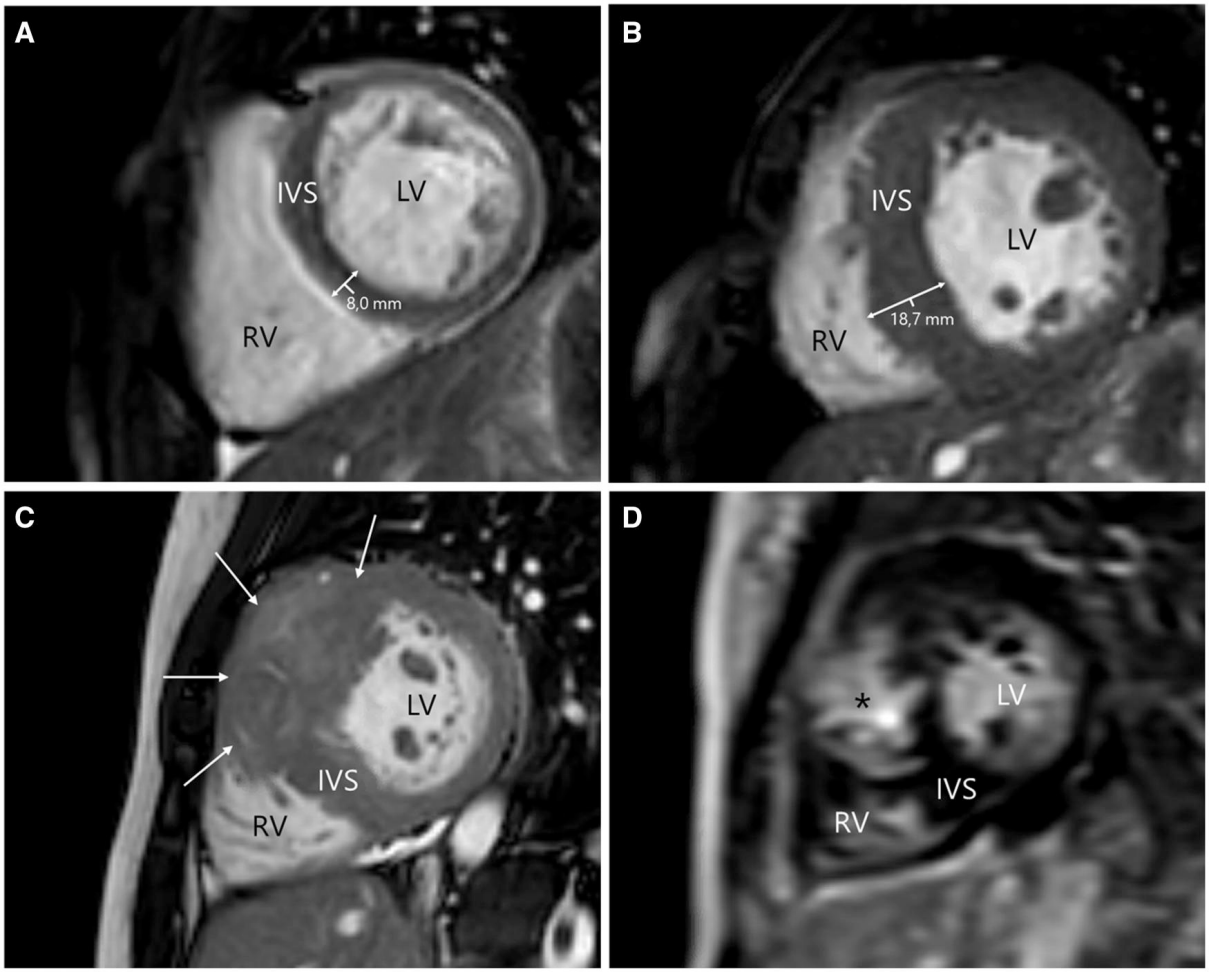
Short-axis CMR images showing left ventricular wall abnormalities in HCM patients. Panel (A) shows a short-axis cine image of a 14-year-old boy demonstrating normal LV wall dimensions (wall thickness of 8 mm). Panel (B) shows a short-axis cine image of a 7-year-old boy demonstrating circumferential LV wall hypertrophy with intraventricular septal wall thickness of 19 mm and LV wall mass of 102 g (110 g/m^2^ indexed for BSA). Reference value for normal LV wall mass for a boy in the 6-12 year age group is 53 g/m^2^ (SD 44-58 g/m^2^).^65^ There was no LGE on the post-contrast images (not shown). Panel (C) shows a short-axis cine image of a 17-year-old girl demonstrating severe asymmetric focal LV wall hypertrophy confined to the anterolateral part of the intraventricular septum (arrows). Panel (D) shows a short-axis delayed enhancement image of the patient from panel (C), demonstrating profound mid-wall LGE in the hypertrophied intraventricular septum wall (asterisk). Abbreviations: CMR = cardiac magnetic resonance, HCM = hypertrophic cardiomyopathy, IVS = interventricular septum, LGE = late-gadolinium enhancement, LV = left ventricle, RV = right ventricle.

Cardiac magnetic resonance imaging sequences allow for accurate assessment of cardiac morphology and function, blood flow quantification, myocardial tissue characterisation, and myocardial perfusion.^26^^,^^27^ The standard imaging modality for evaluation of the cardiac morphology and function is cine-CMR imaging, which provides quantification of cardiac parameters such as atrial and ventricular volumes, myocardial mass, wall motion, and ejection fraction.^28^^,^^29^ Cine sequences, based on steady-state free-precession sequences, are not subject to acoustic window limitations of the heart as they can offer multiplanar imaging with complete coverage of the entire myocardium.^20^^,^^28^ The high blood-myocardial contrast resolution of cine-CMR allows for precise delineation of the endocardial border, so myocardial wall thickness can be assessed and a differential diagnosis of non-compaction can be excluded (Figure 1A-C).^24^^,^^26^^,^^30^ Phase-contrast sequences can be used to perform flow quantification of aortic and mitral insufficiency.^31^

Assessment of myocardial replacement-fibrosis and myocardial tissue characterisation can be performed with contrast-enhanced sequences like late-gadolinium enhancement (LGE) and parametric myocardial sequences such as T1 and T2 mapping.^32^ LGE imaging sequences allow for the identification of focal areas of myocardial fibrosis, a histopathologic characteristic typically seen in the interventricular septum of HCM patients (Figure 1D).^9^^,^^17^ The LGE technique is based on the intravenous administration of gadolinium, with an extracellular and extravascular distribution pattern. Fibrotic tissue—with an increased extracellular volume (ECV) compared to the healthy myocardium it replaced—will be displayed as an enhanced area when compared to the adjacent viable myocardium, as gadolinium takes longer to enter through the expanded extracellular space with a prolonged clearance in scarred areas. LGE image acquisition following gadolinium administration therefore allows for precise identification of the location and extent of the myocardial fibrotic tissue.^5^^,^^29^^,^^33^^,^^34^ Parametric mapping is an alternative myocardial tissue characterisation technique, based on changes in the (myocardial) T1 relaxation times without the need of a contrast agent.^32^ T1 mapping sequences can be used for the detection of diffuse and interstitial myocardial fibrosis, as native T1 (pre-contrast) values of the myocardium are increased in these regions compared to healthy myocardium.^5^^,^^35^ Moreover, the ECV can be calculated using pre- and post-contrast T1 mapping to assess the extent of myocardial fibrosis. Native T1 and ECV are both representatives of extracellular matrix expansion, which is seen in the presence of interstitial fibrosis.^36^^,^^37^ HCM patients are characterised by prolonged T1 relaxation times and native T1, together with ECV expansion.^37^ Parametric mapping for diffuse interstitial fibrosis is therefore complementary to LGE, which allows for the identification of focal areas of myocardial fibrosis.

Perfusion defects can occur in HCM patients particularly in the hypertrophied myocardial segments, which can be evaluated with myocardial perfusion CMR sequences.^38^^,^^39^ First-pass perfusion CMR uses the dynamic inflow of a contrast agent into the myocardium allowing for quantification of the myocardial blood flow along with the myocardial perfusion reserve as well as the visualisation of perfusion defects, either at rest or during pharmacological stress.^39^^,^^40^

## Diagnostic value of CMR in childhood HCM

In recent years, CMR is increasingly used in the diagnostic assessment of children with established or suspected HCM. The ability of CMR to detect subtle abnormalities in myocardial anatomy makes it superior to other imaging modalities in identifying the heterogeneous phenotypic expressions of HCM.^20^^,^^41^ Correct identification of the individual HCM phenotype is of importance for the determination of the specific underlying aetiology, as this is likely to have a significant impact on the patient’s disease prognosis and survival.^3^

According to the 2020 American Heart Association/American College of Cardiology (AHA/ACC) Guideline for the Diagnosis and Treatment of Patients with HCM, the phenotypical diagnosis of childhood HCM is based on the degree and extent of LV hypertrophy (LVH) in the absence of abnormal loading conditions. LVH is defined as an increased LV wall thickness of more than 2 standard deviations greater than the predicted mean for body surface area in children with a family history of HCM or a positive genetic test, or an increased LV wall thickness of more than 2.5 standard deviations in children without a positive family history.^1^ Nearly any pattern and distribution of LVH can be observed in HCM, resulting in various types of HCM depending on the location of the thickened myocardium. The most common presentation of HCM is characterised by LVH at the basal anterior septum in continuity with the anterior free wall.^29^^,^^42^^,^^43^ CMR allows identification of the location and extent of these regional areas of LVH which are not easily identified by echocardiography, particularly at the level of the lateral or basal anterior wall of the LV and the apex.^23^^,^^27^ Therefore, CMR contributes to obtaining correct and differential diagnosis when assessing the location and extent of hypertrophy in patients, using its multiplanar imaging of the entire myocardium.

Furthermore, the presence and location of focal and/or diffuse fibrosis displayed with CMR tissue characterisation are of additive value for HCM diagnosis. Paediatric HCM patients show similar LGE patterns as those seen in adults with HCM,^17^ typically a patchy mid-wall distribution located at the interventricular septum or in the segments with hypertrophy (Figure 1D).^23^^,^^42^^,^^44^ Increased native T1 values and expansion of the ECV are seen in paediatric HCM patients as an expression of focal myocardial fibrosis.^37^^,^^42^ Impaired myocardial perfusion can be observed in affected myocardial segments in the absence of coronary artery disease.^38^

Besides unravelling the specific HCM phenotype, the previous mentioned diagnostic CMR values aid to differentiate HCM from alternative causes of LVH, such as an athlete’s heart or a storage disease.^1^^,^^8^ The presence of fibrotic tissue by LGE and/or T1 mapping techniques supports the diagnosis of HCM in patients with LVH.^5^^,^^44^

## Prognostic value of CMR in childhood HCM

As mentioned previously, SCD is the leading cause of death in childhood HCM.^3^^,^^13^ Besides SCD, childhood-onset HCM is associated with higher mortality risk and poorer long-term outcomes compared to adult-onset HCM.^5^^,^^9^^,^^14^ Therefore, it is crucial to obtain an accurate prognostic stratification, as the outcome varies greatly depending on the underlying cause and age at diagnosis,^4^ and will have a potential impact on the child’s long-term quality of life.

The ability of CMR to characterise the degree and extent of LVH and to delineate myocardial fibrosis is of prognostic value for the evaluation of risk stratification in adult HCM and potentially also in childhood HCM.^17^^,^^27^ The magnitude of LVH is strongly linked with an increased risk of SCD in childhood HCM. Severe LVH, expressed by myocardial areas with a score of 6 standard deviations above the predicted mean for body surface area, is considered a major clinical risk factor for potentially life-threatening ventricular arrhythmias.^17^^,^^19^

Multiple studies looked at the predictive value of LGE (on CMR) as an important risk factor for the occurrence of ventricular arrhythmias and SCD in childhood HCM.^9^^,^^17^^,^^43^^,^^45^^,^^46^ Their data showed an increased risk of adverse events in HCM patients when LGE is present compared to those without the presence of LGE, as areas of fibrosis often constitute the substrate for the triggering of ventricular arrhythmias at the basis of SCD. Moreover, the extent of LGE is an important risk factor for ventricular arrhythmias.^1^ Extensive or severe LGE is defined as an extension of LGE greater than 15% of the total amount of left ventricular myocardial mass.^9^^,^^26^^,^^47^ In childhood HCM, LGE is most prominent in patients with more extensive hypertrophy, emphasising the close relationship between LGE and the extent of LVH.^9^

Reduced myocardial perfusion is seen in HCM patients, particularly in the endocardium with the highest wall stress.^48^ The degree of reduced perfusion is proportional to the magnitude of hypertrophy and the extent of fibrosis.^39^ Reduced myocardial perfusion is associated with adverse LV remodelling and an impaired vasodilatory capacity, known as microvascular dysfunction, which is a predisposing factor for myocardial ischemia.^38^^,^^40^ Therefore, the extent of microvascular dysfunction and/or myocardial ischaemia in HCM has been associated with poor clinical outcomes, as it worsens with increasing HCM disease severity.^39^ Besides, abnormal perfusion has been observed in both hypertrophied and non-hypertrophied myocardial segments, suggesting it may occur early in the disease manifestation and might be useful as a prognostic predictor.^39^^,^^49^

At present, there is limited data on the prognostic role of T1 mapping in childhood HCM.^50–52^ In adult HCM, the role of T1 mapping and thus the risk stratification of the diffuse myocardial fibrosis points towards a prognostic significance, particularly for higher ECV as an independent risk marker.^53^ Therefore, T1 mapping might also be an interesting outcome predictor for paediatric HCM patients and further investigations are needed for the development of clinical implementation.

Together, these prognostic markers contribute to the prognostic stratification and sustain an expanded role of CMR in the clinical assessment of paediatric patients with HCM. Based on the prognostic markers, strategies can be established to predict the development and management of the disease. Clinical management of children with HCM is mainly focused on reduction and alleviation of symptoms, prevention of disease-related complications and slowing down the progression of the disease, all to improve the patient’s quality of life.^4^^,^^54^

## CMR in clinical follow-up

Childhood HCM can be progressive over time. The rate, timing and degree of disease progression vary from one patient to the other, therefore close monitoring and serial follow-up throughout the life of the diagnosed patients is of paramount importance.^29^ Given this, the diagnostic and prognostic features of CMR for the implementation of clinical evaluation of patients over time are of added value and may lead to modifications in the long-term prognosis and adaptations to clinical management approaches of individual patients.^23^

The 2020 AHA/ACC guidelines recommend repeated CMR imaging periodically in adult HCM patients every 3 to 5 years for the purpose of SCD risk stratification.^1^ However, nothing is mentioned about repeated CMR imaging in paediatric HCM patients. At present, echocardiography is the only recommended technique for screening paediatric HCM patients during follow-up.^1^ As childhood is known to be a time of significant HCM development and progression, it is likely that the varying diagnostic CMR values reflect a changing cardiac phenotype over time.^55^ The increased development and progression of hypertrophy of the LV myocardium in paediatric HCM patients is well-known.^56^ In addition, Axelsson-Raja *et al* and Ali *et al* have described the progressive nature of myocardial fibrosis in paediatric HCM patients using LGE in follow-up CMR.^45^^,^^46^ They defined an increase in the presence and proportion of LGE in patients during serial CMR imaging. Hence, paediatric HCM patients, especially those with LGE present at the time of diagnosis, may also benefit from periodic CMR imaging, as myocardial fibrosis progresses over time. To determine clinical changes over time, it is suggested by Axelsson-Raja *et al* to perform periodic screening every 1 to 3 years during serial follow-up.^45^

## CMR for family screening

As stated before, HCM is a heritable disease that can manifest at any age.^1^ Therefore, it is equally important to clinical (and genetic) screen first-degree relatives of the affected patient to identify at-risk family members with HCM. The purpose of family screening is to identify a relative with HCM earlier in life. Early diagnosis is valuable as it enables initiation of early treatment, prevention strategies and takes a closer look at the clinical surveillance, but also the potential to prevent or mitigate major cardiac events.^57^ In addition, especially young family members should be considered for early screening, as the phenotype of HCM is varied and includes a higher risk of adverse outcomes when diagnosed during childhood.^4^^,^^55^^,^^58^ This early screening is in line with the current 2020 AHA/ACC guidelines, which endorses to clinically (and genetically) screen younger at-risk family members at any age, instead of starting from the previously recommended age of ten years.^1^^,^^59^ Also, implementation of repeated clinical assessment is required throughout child- and adulthood.^1^ As family members displaying a normal clinical cardiac evaluation should not necessarily be assumed to be free of risk, due to the possibility of subsequent development of HCM.^60^

Similar to serial follow-up of HCM patients, echocardiography is the recommended technique to perform cardiac screening and follow-up of first-degree family members.^1^ While this is still the case, CMR images are superior to echocardiography in acquiring detailed morphological assessment of myocardial hypertrophy.^3^ Furthermore, CMR contains additional advanced techniques to characterise the myocardial tissue and determine the presence of myocardial fibrosis, which is not possible with echocardiography. With its sharp contrast and high spatial features, CMR measurements show high accuracy and reproducibility which allows for precise comparisons to be made over time.^17^ Therefore, to monitor the progressive nature of paediatric HCM patients and their relatives, the technical features of CMR are favourable compared to echocardiography and could be considered the preferred imaging method throughout long-term follow-up.

## Future perspectives

At this moment, a lot of the diagnostic, prognostic and risk stratification models for children with HCM are largely extrapolated from adult HCM criteria, especially for the implementation of CMR findings.^1^^,^^58^ To unravel the specific paediatric characteristics, to establish paediatric reference values and to provide standardisation of the application of CMR in children, more research is required. Currently, there is a shortage of (universal) paediatric normative datasets, like parametric mapping and tissue characterisation.^61^ T1 mapping results vary between the different vendors and types of CMR scanners (including the magnetic field strength and the manufacturer). Therefore, local reference T1 values are typically used by individual institutions to identify abnormal myocardium.^32^ The resulting difficulty of interpreting and comparing paediatric CMR studies highlights the need of standardisation. Other emerging CMR techniques could be used in paediatric HCM patients, like strain measurements, 4D flow and diffusion tensor imaging and evaluation of the atria.^61^^,^^62^ However, the clinical relevance and applicability of these techniques in the paediatric population should be further explored first. Subsequently, LGE and perfusion imaging techniques could be further improved by implementing (deep learning based) quantitative approaches. Quantification may increase accuracy and reproducibility facilitating clinical follow-up and decision making.^63^^,^^64^

Overall, formulation of uniform clinical definitions of diagnostic and prognostic conditions of CMR findings will provide more consistent imaging protocols, which will make it easier to combine and compare CMR findings in childhood HCM. Additionally, these paediatric characteristics provide the implication of CMR into clinical follow-up, to predict and reassess a patient’s risk, taking the changing cardiac phenotype into account. It would be of added value to obtain a clinical CMR baseline for each patient with childhood HCM, as each heart and patient is unique. Personalised clinical evaluation, management and investigation can be implemented as a direct result of this. Follow-up CMR can be applied in patients with more advanced disease characteristics at their CMR baseline, or when progressive HCM appears over time.

## Conclusion

Cardiac magnetic resonance has established its clinical role in paediatric patients with suspected or established HCM. The comprehensive features of CMR provide an accurate cardiac assessment, making CMR suitable for the identification of the disease aetiology and the establishment of a patients’ individual prognostic risk factors. Besides morphological and functional assessments, CMR has the unique ability to characterise the composition and perfusion of the myocardium. CMR is able to visualise and quantify myocardial-replacement fibrosis, using LGE sequences for focal fibrosis and parametric mapping for diffuse fibrosis. The patient-specific cardiac information and risk stratification acquired through CMR is beneficial in the acquisition of early diagnosis and consequently in improved long-term survival. This is of particular importance since childhood HCM is a progressive disease and associated with an increased risk of life-threatening ventricular arrhythmias and SCD, compared to the adult HCM population. Taken together, CMR has shown to be of additional value in the evaluation of childhood HCM patients, as it provides proper clinical information for diagnosis, prognosis, and serial follow-up.
